# Headache yesterday in Europe

**DOI:** 10.1186/1129-2377-15-33

**Published:** 2014-05-28

**Authors:** Colette Andrée, Timothy J Steiner, Jessica Barré, Zaza Katsarava, Jose Miguel Lainez, Christian Lampl, Michel Lantéri-Minet, Daiva Rastenyte, Elena Ruiz de la Torre, Cristina Tassorelli, Lars Jacob Stovner

**Affiliations:** 1 Center of Public Health Research, CRP-Santé, Strassen, Luxembourg; 2 Department of Pharmaceutical Sciences, University of Basel, Basel, Switzerland; 3 Department of Neuroscience, Norwegian University of Science and Technology, Trondheim, Norway; 4 Division of Brain Sciences, Imperial College London, London, UK; 5 Department of Neurology, University of Essen, Essen, Germany; 6 Department of Neurology, Hospital Clinico Universitario, University of Valencia, Valencia, Spain; 7 Headache Medical Center Seilerstaette Linz, Department of Neurogeriatric Medicine and Remobilisation, Hospital of the Sisters of Charity, Linz Seilerstaette 4, 4010 Linz, Austria; 8 Département d’Evaluation et Traitement de la Douleur, Centre Hospitalo-Universitaire de Nice, Nice, France; 9 Lithuanian University of Health Sciences, Kaunas, Lithuania; 10 Asociación Española de Pacientes con Cefalea (AEPAC), Valencia, Spain; 11 Centro Italiano di Ricerche Neurologiche Applicate (CIRNA) and Headache Science Centre, National Neurological Research Institute C. Mondino Foundation, University of Pavia, Pavia, Italy; 12 Norwegian National Headache Centre, St. Olavs University Hospital, Trondheim, Norway

**Keywords:** Headache, Impact, Cost of illness, Prevalence, Eurolight project, Global Campaign against Headache

## Abstract

**Background:**

Surveys enquiring about burden of headache over a prior period of time (*eg*, 3 months) are subject to recall bias. To eliminate this as far as possible, we focused on presence and impact of headache on the preceding day (“headache yesterday”).

**Methods:**

Adults (18-65 years) were surveyed from the general populations of Germany, Italy, Lithuania, Luxembourg and the Netherlands, from a work-force population in Spain and from mostly non-headache patient populations of Austria, France and UK. A study of non-responders in some countries allowed detection of potential participation bias where initial participation rates were low.

**Results:**

Participation rates varied between 11% and 59% (mean 27%). Non-responder studies suggested that, because of participation bias, headache prevalence might be overestimated in initial responders by up to 2% (absolute). Across all countries, 1,422 of 8,271 participants (15-17%, depending on correction for participation bias) had headache yesterday lasting on average for 6 hours. It was bad or very bad in 56% of cases and caused absence from work or school in 6%. Among those who worked despite headache, 20% reported productivity reduced by >50%. Social activities were lost by 24%. Women (21%) were more likely than men (12%) to have headache yesterday, but impact was similar in the two genders.

**Conclusions:**

With recall biases avoided, our findings indicate that headache costs at least 0.7% of working capacity in Europe. This calculation takes into account that most of those who missed work could make up for this later, which, however, means that leisure and social activities are even more influenced by headache.

## Background

The World Health Organization (WHO) has acknowledged headache disorders as of global public-health importance [1]. This is good for people with headache, who carry most of its burden but who find that it receives little priority in the queue for health care [1]. Headache disorders are under-diagnosed and mostly undertreated [1,2], not because diagnosis is particularly difficult or because effective treatments do not exist but because of widespread failure of health services to recognize the need for health care for headache and to take steps to deliver it [1,2]. In these circumstances, the recent Global Burden of Disease Survey 2010 (GBD2010) [3] found tension-type headache (TTH) and migraine to be the second and third most prevalent disorders in the world, and migraine the seventh highest specific cause of disability [4].

In fact there are large gaps in our knowledge of the prevalence and burden of headache worldwide [5,6]. In Europe, knowledge is most complete in western countries, yet focuses strongly on migraine; rather few studies have addressed the more prevalent TTH or the more disabling headache disorders characterized by headache on ≥15 days/month. Countries to the east, particularly those of the former USSR, have until recently been badly neglected.

The Eurolight project was a collaboration of 25 partners from 15 countries in Europe [7] supported by the EC European Agency for Health and Consumers. Eurolight’s main objective was to bolster the political argument for better investment in health-care services for people with headache throughout Europe. For this purpose, it sought to adduce evidence of the impact of migraine, TTH and headache on ≥15 days/month across the EU, and achieved this by conducting surveys in ten countries. It added estimates of impact to those of prevalence [8], thereby filling an important part of the knowledge gap in Europe, and showed, among other large burdens, the enormous financial costs (over €100 billion annually) imposed by headache disorders [9].

Burden-of-headache studies generally estimate 1-year prevalence and enquire into quantifiable components of burden (for example, symptom burden, disability, time loss, financial loss, impact on quality of life) through questionnaires that depend upon recall over a preceding period (commonly 3 months). It is not known how reliable recall is for such matters, but at best it is likely to be variable and at worst highly misleading [10]. Recent studies initiated by the Global Campaign against Headache [5] in Russia [11], China [12,13], India [14], Pakistan [15] and Zambia (unpublished) introduced enquiry into “headache yesterday”, and Eurolight followed this lead. In such an enquiry, numbers responding positively are obviously reduced: the prevalence of headache yesterday is substantially lower than the 1-year prevalence, given that headache-affected days vary in frequency between individuals with headache from one to 365 per year. On the other hand, responses to enquiries about headache yesterday are not subject to recall bias.

The full methodology [7,16] and principal results [8] of Eurolight are published elsewhere. Here are presented the results on headache yesterday.

## Methods

Eurolight was essentially an EU-wide study employing a modified and pragmatic form of cluster sampling to reach a large number of participants in many countries. Questionnaire-based surveys were conducted in 10 countries: Austria, France, Germany, Ireland, Italy, Lithuania, Luxembourg, Netherlands, Spain and United Kingdom (UK).

### Ethics

The National Ethics Committee of Luxembourg gave overall approval of the protocol. Further approvals were obtained from national or local ethics committees wherever needed as the methods for recruitment of participants differed between countries. Similarly, data protection approvals were obtained centrally in Luxembourg and at country levels in compliance with national and European privacy laws.

In each country, prospective participants received a written information sheet explaining the project and enquiry, and their purpose.

### Questionnaire

The development, content and validation of the structured questionnaire, and its translation into all local languages, have been previously described [16]. Demographic, screening (for headache) and headache-diagnostic questions (based on ICHD-II [17]) were supplemented by several question sets addressing impact. Duration of headache yesterday was recorded categorically (<1, 1-4, 5-12 or >12 hours). Intensity of headache yesterday was graded on a verbal rating scale (“not bad”, “bad” or “very bad”, these being terms that lent themselves better to translation than the customary “mild”, “moderate” or “severe”). Absence from work or school yesterday because of headache was recorded in three categories (“less than half” or “more than half the day” or “all day”). In those who worked yesterday despite headache, two further questions enquired into functional impairment: the first asked how much, of that expected to be done, was actually done (“everything”, “more than half”, “less than half” or “nothing”); the second asked whether any shortfall would be made up for later (“yes” or “no”). Final questions were about household chores and planned social activities actually done yesterday despite headache (“everything”, “more than half”, “less than half” or “nothing” in either case).

### Sampling and data collection

Methods varied between countries according to what was feasible. Again, these are fully described elsewhere [7]. Table 1 provides a summary. The sample drawn from Lithuania was fully population-based. In other countries, samples were to varying degrees less population-based.

**Table 1:** Summary of data collection methods in each country

Country*	**Sample size (n)**	**Methods**
Lithuania	1,137	Sample drawn from inhabitants of Kaunas city and Kaunas region using Residents’ Register Service, reflecting age (in range 18-65 years) and gender composition of Lithuania and proportions living in rural (33%) or urban (67%) areas. Data collection face-to-face, conducted by medical students “cold-calling” door-to-door.
Luxembourg	6,498	Sample aged 18-65 years, stratified for age, gender, region and nationality, drawn from general population via national social security registry (IGSS). Questionnaires distributed and returned by post. Reminders sent one month later to non-responders.
Spain	1,700	Random sample of employees of various companies operating in national postal services in 10 areas of Spain, stratified to be representative of general working population with regard to gender, age (within range 18-65 years) and education. Ten occupational health physicians delivered and took return of questionnaires. One reminder by telephone to non-responders.
Germany	3,000	Random urban (50%) and rural (50%) samples aged 18-65 years from general population listings supplied by local municipal authority. Questionnaires distributed and returned by post. No reminders sent.
Italy	3,500	Random urban (70%) and rural (30%) samples drawn from general population using listings supplied by Azienda Sanitaria Locale of Pavia, stratified with regard to gender, age (in range 18-65 years) and education. Questionnaires distributed and returned by post. No reminders sent.
France	2,400	Consecutive patients aged 18-65 years attending any of cooperative of 80 general practitioners (GPs) on a pre-specified day. Questionnaires to be completed and returned immediately or later by post. One reminder by email after one week to non-responders.
Austria	up to 6,000	Up to 10 consecutive patients aged 18-65 years visiting any of 400 GPs and 200 neurologists for any reason on a pre-specified day. Questionnaires to be completed and returned later. One reminder after one month to non-responders.
Netherlands	unknown	Survey conducted by TNS-NIPO, a market research company with access to a population sample of 200,000, representative with regard to gender, age (in range 18-65 years), region and education. Questionnaire distributed by internet, to be completed on-line. Study stopped when >2,000 received back.
UK	720	Modified population-based sampling attempted through 12 GP practices in 11 areas (in UK, virtually all residents are registered with local GP). Questionnaire given to consecutive patients aged 18-65 years attending for any reason over a period of time, to be completed and returned immediately, or later by post.

*Countries are listed in descending order corresponding to how well the sample was population-based.

Additional surveys in Spain and the Netherlands, and the only survey in Ireland, were performed among members of the national headache patients’ organizations. The samples generated from these were inevitably biased [7] and the data from them are not used here. Consequently, only nine countries contributed to the analysis.

### The non-responder study

In all countries, questionnaires were more likely to be completed and returned by those most affected by headache (participation bias), and thus would not be representative of the initial samples (particularly relevant because of the rather low participation rates in some countries). Therefore, studies of non-responders were performed in Luxembourg (n = 357), Italy (n = 202), Netherlands (n = 188) and Germany (n = 260). In Italy, non-responders were invited by means of advertisements in local newspapers to complete a short questionnaire on the website of Centro Italiano di Ricerche Neurologiche Applicate (CIRNA) (http://www.cefalea.it). In the other countries, non-responders were selected randomly and called by telephone. All were asked whether headache had occurred ever (yes or no), during the last year (and how often), and yesterday (yes or no).

### Data management and analysis

All completed questionnaires were transferred electronically to the data-management centre at CRP-Santé. Double-data-entry and reconciliation of inconsistencies were employed as quality-control procedures.

Statistical analyses were performed at CRP-Santé using SAS version 9.2, and the further calculations of results described in the text were performed by LJS, using Excel version 14.0.6123.5001. Many analyses were purely descriptive. Not all participants answered the questions on headache yesterday; to determine the prevalence of headache yesterday, the denominator was taken as all those who returned questionnaires, with or without these questions answered, which gave a more conservative estimate. Prevalence estimates were adjusted for gender imbalance: since more women than men returned questionnaires, the overall prevalence was calculated as the weighted mean of the prevalences estimated separately for men and women.

## Results

**Figure 1:**
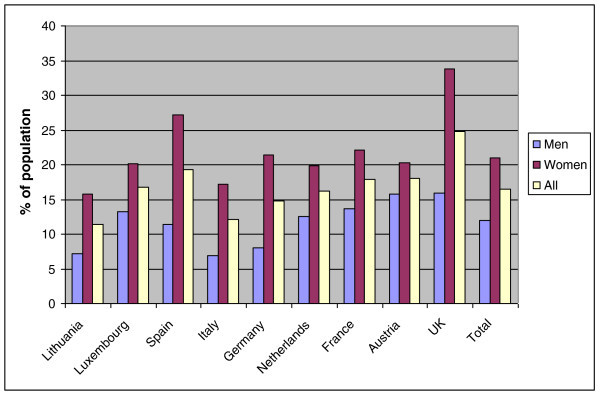
Prevalence of headache yesterday in nine European countries.

In nine countries, 8,271 participants not derived from specific headache-patient populations (*ie*, excluding the patient-organization samples from the Netherlands, Spain and Ireland), participated in the survey. A majority (58%) were female. For each country, the number of participants and their gender are shown in Table 2. The overall participation rate in the study (excepting the Netherlands, where the denominator was indeterminable, and Austria, where it was not recorded) was 27% (details are reported elsewhere [7]).Altogether, 1,422 participants (17.2%) reported headache yesterday. The prevalences of headache yesterday in the nine countries were rather constant in the main study, ranging from 11% in Lithuania to 19% in Spain (gender-adjusted mean: 17%; SD: 0.4%), with the UK, which had very low numbers, appearing as an outlier (25%) (Figure 1). In the non-responder study, with 1,007 participants, there were 91 (9.0%) with headache yesterday.

**Table 2:** Numbers and gender of participants by country

**Country**	**Participants (n)**	**Male: female (%)**
Austria	644	30:70
France	876	32:68
Germany	318	43:57
Italy	487	42:58
Lithuania	573	41:59
Luxembourg	1,833	41:59
Netherlands	2,414	50:50
Spain	999	41:59
United Kingdom	127	35:65
Overall	8,271	42:58

Most participants (47%) reported a duration of headache yesterday of 1-4 hours, which might reflect effect of treatment; 24% reported 5-12 hours and 15% reported >12 hours. The estimate of the overall mean (taking the mid-point of each range) was 6 hours (Table 3). In all samples, most headache yesterday was reported as “not bad” (overall mean 44%) or “bad” (overall mean 44%); even so, headache yesterday was “very bad” in 11%.

**Table 3:** Prevalence, duration and impact of headache yesterday in nine European countries

	**Overall**	**Males**	**Females**
Headache yesterday (HY) (% [CI])	17 [16-18]	12 [11-13]	21 [20-22]
(n/N of participants in the study)	1,422/8,272	414/3,456	1,007/4,791
Duration of HY (median) (hr)	6	5	6
Intensity of HY (“bad” or “very bad”) (%)	56	52	58
Functional impairment during HY (severely affected or could do nothing) (%)	34	32	35
Lost all day at work or school because of HY (% [CI])	6 [4-8]	7 [4-11]	5 [4-7]
(n/N of participants for whom yesterday was a workday)	41/726	15/217	26/509
Worked with HY, but did nothing or < half of expected (% [CI])	20 [17-23]	18 [13-24]	20 [17-24]
(n/N of participants who worked with HY)	134/683	36/204	98/479
Lost all day from household chores because of HY (% [CI])	11 [10-13]	13 [10-16]	11 [9-13]
(n/N of participants responding)	167/1,453*	53/415*	114/1,038*
Lost all social activities yesterday because of HY (% [CI])	24 [22-27]	22 [18-26]	25 [23-28]
(n/N of participants responding)	351/1,442*	90/416*	261/1,026*

CI: 95% confidence interval.

*some people answered this question although they had not answered “yes” to the question about headache yesterday (numerator n in first row of the table).

**Figure 2:**
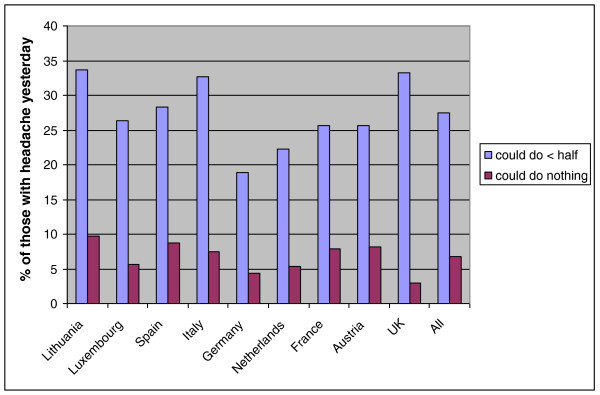
Percent of people with severely impaired working capacity among those with headache yesterday in nine European countries.

Functional impairment yesterday because of headache showed that most people (72%) were adversely affected to some extent, while about a quarter (overall mean 27%) were severely affected (could do less than half of what they expected) and a minority (overall mean 7%) could do nothing (Table 3). Variation between countries was not enormous (Figure 2). Yesterday was a workday for 51% of those with headache yesterday. Absence rates from work were more variable but, again overall, 6% of those with headache yesterday (or 0.5% of all participants) lost the entire day. Of those who worked yesterday despite headache, 20% did less than half or nothing of what they expected, and only a small majority (55%, somewhat more than the 28% reporting normal function) responded that they actually did everything. Of those who did less, the majority (71%) claimed they would be able to make up for it later. The pattern was somewhat different for household chores, with fewer people (36%) achieving everything expected and more (11%) doing nothing, and for social activities (33% achieving everything, 24% doing nothing).

The prevalence of headache yesterday was much higher in women than in men (21% *vs* 12%). However, with regard to headache duration and intensity and the measures of functional impairments, men and women were quite similar (Table 3).

## Discussion

The importance of measuring the impact of headache yesterday, and the major strength of this study, lies in the freedom from recall bias. This form of bias potentially affects all studies retrospectively recording impact over a period of time, to an unknowable degree. We emphasise the distinction between recall *error* and recall *bias*. Recall of what happened *yesterday* may not be error-free, but the error is likely to be neither systematic nor large enough that it might lead to bias. There may be exaggeration, or mis-description in other ways, but these are not forms of recall bias, and they occur in any enquiry.

What happened yesterday is not, of course, indicative of the impact of headache in most individuals, but at *population* level it is. Numbers of participants contributing data are much reduced when the focus is on headache yesterday, but this is countered effectively here by the large original numbers in this study. The impact of headache yesterday therefore, we believe, robustly and accurately describes the impact of headache overall.

The collection of data from multiple samples drawn from nine countries in Europe yielded results relevant more to Europe as a whole than to the individual countries. This was the intention. We have presented some limited graphical comparisons between countries, but emphasise that there are methodological limitations inherent in a study of this sort. Not all samples were population-based, although differences that might be attributable to this were, perhaps surprisingly, not very apparent: variations between countries were not unduly large. Nevertheless, our data should *not* be used to make comparisons between the nine countries because of the differences in sampling and data collection methods in each, and, importantly, the relatively small sample sizes in some of them.

Although diagnostic questions were included in the methods, the responses to these are not analysed here. This is mostly because these questions were applied to the subjectively most bothersome headache reported in the last year, not to headache yesterday. Although we asked whether headache yesterday was of the same type as the most bothersome, and in over two thirds (69%) of cases it was, there was no diagnosis made in the remaining 31%. This is not important to our message, which is a public-health one: headache disorders, without focus on migraine, are a major cause of population ill-health, representing a need for which health services must make due provision.

A potential source of bias was headache *today*: participants with headache on the day they encountered the questionnaire might have deferred responding until they became headache-free, and then been highly likely to report headache yesterday. Headache today is as probable as headache yesterday; therefore, it might seem that this factor could double the reported prevalence of headache yesterday. In reality this was not so, because about half of the 1-day prevalence of headache is attributable to daily headache (the probability of episodic headache yesterday is <7% [*ie*, 2/30] of its long-term prevalence, assuming an average frequency of 2 days/month, whereas the probability of daily or near-daily headache being present yesterday is close to 100% of its prevalence). When headache occurs daily, it is of no consequence whether the enquiry is conducted today or tomorrow. The problem would not have occurred in Lithuania, where participants were visited without prior notice, and did not have the option of deferring their responses until tomorrow. The reported prevalence of headache yesterday was lowest in this country, but not outlying (Figure 1).

Low participation rates did inject bias. The purpose of the non-responder study was to detect participation bias, which, according to this study, led to an over-estimation of the prevalence of headache yesterday. However, the non-responder study had relatively few numbers, and a much less-detailed enquiry; the data from it were of lower quality than those from the main study. It would be quite inappropriate to substitute the former for the latter; rather, the former might be considered to demarcate minima. Responders in the main study, of whom 79% reported headache last year, were 27% of the surveyed sample (this being the participation rate); the non-responder study therefore represented the other 73%, of whom 64% (gender-adjusted) had headache last year [8]. The weighted average of 68% ([79*27 + 64*73]/100) establishes the minimum 1-year prevalence. We assumed that headache yesterday could occur only in those reporting headache last year, and adjusted on this basis. In the continuing discussion below, ranges are presented that reflect this.

An estimated 15-17% (the lower limit = 17*68/79) of adults aged 18-65 years in Europe had headache yesterday, which implies a similar percentage *every* day and signals a huge level of population ill-health. This range should be internally consistent with the approximate estimate derived from the 1-year prevalence (78.6% [8]) and average frequency (5.8 headache days/month [unpublished]) in the same population. These data generate a mean of 459 headache days per month per 100 of the population, from which it can easily be calculated that 15% of the population have headache on any day (13% when potential participation bias is considered). These figures are reassuringly similar, despite being subject to recall bias. Few other data exist on headache on any single day; there is one recent study from mainland China specifically on headache yesterday, reporting an age-adjusted prevalence of 4.8% in the general population aged 18-65 years [13]. This is a little less than one third of our finding, but the estimated 1-year prevalence of headache in China (23.8% [18]) is also about a third of that in Europe [14]. Our finding of 15-17% for headache yesterday in Europe is consistent with the point prevalences (16 and 19%) found in two carefully conducted Danish studies [19,20].

The purpose of reporting headache yesterday was, however, to focus not on prevalence but on impact. Duration and intensity are dimensions of symptom burden; whilst they may be misleading because they are reported subject to the effects of any treatments taken, they give rise to the following estimation. If 15-17% of a population had headache yesterday of average duration 6 hours, then one quarter of these (say 4.0%) had headache at any particular moment (which compares with 1.8% reported in China [13]). Headache was bad or very bad in 56% of those with headache, or 2.2% of the population aged 18-65 years (1.4% in China [13]). Taking the population of the EU in the age range 18-65 years as 300 million (http://europa.eu/about-eu/facts-figures/living/), we calculate that, at this and any moment, between 6.3 (300*0.56*0.15/4) and 7.1 (300*0.56*0.17/4) million people are suffering in Europe with bad or very bad headache.

As telling are the secondary effects – in particular, functional impairment and its consequences. Thus, an estimated 0.4 (0.5*68/79) to 0.5% of working-age adults in Europe lost the whole of yesterday at work because of headache, again implying a similar percentage every (working) day. A further 1.2-1.6%, working despite headache yesterday, reported a significant level of inefficiency (less than half or nothing achieved). In line with the theory behind the MIDAS instrument [21] on which our lost-productivity questions were based, we counted these also as wholly lost days: the overestimate arising from this was offset by discounting days when >50% but <100% was achieved. Adding these generates a range-estimate of 1.8-2.1% as the total lost productivity at work due to headache yesterday (and every working day). This is not so very different from the 1.3% reported in China [13], despite the much lower prevalence there, reflecting a higher proportion in China reporting disablingly bad headache. Furthermore, in our study, 72% of participants working with impaired efficiency believed they could make up for it later. If so, a reduction by this factor should be applied to this element of lost time (now 0.3-0.4%), yielding a revised total of 0.7-0.9%. It is actually questionable whether “making up later” can achieve complete restitution, but clearly there must be some degree of offsetting. In other words, at least 0.7% of the available working capacity in Europe, and possibly 2-3 times this, is surrendered to headache, which opens a window upon a vista that employers and governments both should find alarming.

The evidently greater negative impact of functional impairment upon household chores and social activities (Table 3) reflects, almost certainly, the more optional nature of these in comparison with duties at work. They are more easily abandoned or reduced, which should not be to underrate their importance as a part of everyday life that headache takes away. Given the number of people with headache yesterday, losses of this sort must be acknowledged because their cumulative impact is very high. And there is more: for the 72% of affected participants who could make up later for impaired efficiency at work, something else would presumably be given up in order to do this. There would remain a possibly immeasurable but not necessarily intangible loss expressed elsewhere, and in all probability this would be of leisure time, already a serious casualty of headache. While lost working time has, in theory at least, a calculable financial consequence (either wages are lost or the employer pays for work not done), how leisure time is valued in monetary terms is a complex subject which we will not attempt to explore. It should nonetheless be noted that any value attached to lost leisure time must be added to that of lost work time when the cost of headache is calculated.

Our data show that the burden of headache is much larger in women than in men, mostly because women have headache more often. During headache, men and women appear to have similar symptom burdens and functional impairment.

## Conclusion

Headache is common, and its impact is high. Assessment in nine countries in Europe based on headache yesterday, and therefore free from recall bias, shows that headache affects 15-17% of adults aged 18-65 years on any day. It removes at least 0.7% (and up to three times this) from workforce capacity. Leisure time and social activities are also serious casualties.

## Competing interests

None of the authors has a conflict of interest relevant to the content of this manuscript. TJS, ZK and LJS are directors and trustees of *Lifting The Burden*, a UK-registered non-governmental organization conducting the Global Campaign against Headache in official relations with the World Health Organization.

## Authors’ contributions

The Eurolight project was conceived by CA and designed by CA, TJS and LJS. The concept of “headache yesterday” was developed by TJS and LJS. Data were acquired by CA, ZK, JML, CL, ML-M, DR, ERdlT, CT and LJS, and analysed and interpreted by JB, TJS, LJS and CA. The manuscript was drafted by TJS and revised for intellectual content and approved in the final version by all authors.
